# Evaluating ChatGPT in Qualitative Thematic Analysis With Human Researchers in the Japanese Clinical Context and Its Cultural Interpretation Challenges: Comparative Qualitative Study

**DOI:** 10.2196/71521

**Published:** 2025-04-24

**Authors:** Kota Sakaguchi, Reiko Sakama, Takashi Watari

**Affiliations:** 1 General Medicine Center Shimane University Hospital Izumo Japan; 2 Department of General Medicine NTT Medical Center Tokyo Japan; 3 Integrated Clinical Education Center Kyoto University Hospital Kyoto Japan

**Keywords:** ChatGPT, large language models, qualitative research, sacred moment(s), thematic analysis

## Abstract

**Background:**

Qualitative research is crucial for understanding the values and beliefs underlying individual experiences, emotions, and behaviors, particularly in social sciences and health care. Traditionally reliant on manual analysis by experienced researchers, this methodology requires significant time and effort. The advent of artificial intelligence (AI) technology, especially large language models such as ChatGPT (OpenAI), holds promise for enhancing qualitative data analysis. However, existing studies have predominantly focused on AI’s application to English-language datasets, leaving its applicability to non-English languages, particularly structurally and contextually complex languages such as Japanese, insufficiently explored.

**Objective:**

This study aims to evaluate the feasibility, strengths, and limitations of ChatGPT-4 in analyzing qualitative Japanese interview data by directly comparing its performance with that of experienced human researchers.

**Methods:**

A comparative qualitative study was conducted to assess the performance of ChatGPT-4 and human researchers in analyzing transcribed Japanese semistructured interviews. The analysis focused on thematic agreement rates, interpretative depth, and ChatGPT’s ability to process culturally nuanced concepts, particularly for descriptive and socio-culturally embedded themes. This study analyzed transcripts from 30 semistructured interviews conducted between February and March 2024 in an urban community hospital (Hospital A) and a rural university hospital (Hospital B) in Japan. Interviews centered on the theme of “sacred moments” and involved health care providers and patients. Transcripts were digitized using NVivo (version 14; Lumivero) and analyzed using ChatGPT-4 with iterative prompts for thematic analysis. The results were compared with a reflexive thematic analysis performed by human researchers. Furthermore, to assess the adaptability and consistency of ChatGPT in qualitative analysis, Charmaz’s grounded theory and Pope’s five-step framework approach were applied.

**Results:**

ChatGPT-4 demonstrated high thematic agreement rates (>80%) with human researchers for descriptive themes such as “personal experience of a sacred moment” and “building relationships.” However, its performance declined for themes requiring deeper cultural and emotional interpretation, such as “difficult to answer, no experience of sacred moments” and “fate.” For these themes, agreement rates were approximately 30%, revealing significant limitations in ChatGPT’s ability to process context-dependent linguistic structures and implicit emotional expressions in Japanese.

**Conclusions:**

ChatGPT-4 demonstrates potential as an auxiliary tool in qualitative research, particularly for efficiently identifying descriptive themes within Japanese-language datasets. However, its limited capacity to interpret cultural and emotional nuances highlights the continued necessity of human expertise in qualitative analysis. These findings emphasize the complementary role of AI-assisted qualitative research and underscore the importance of further advancements in AI models tailored to non-English linguistic and cultural contexts. Future research should explore strategies to enhance AI’s interpretability, expand multilingual training datasets, and assess the applicability of emerging AI models in diverse cultural settings. In addition, ethical and legal considerations in AI-driven qualitative analysis require continued scrutiny.

## Introduction

Qualitative research methods are well established across various fields, including social sciences and health care, as a vital approach to understanding complex phenomena [1,2]. Qualitative research aims to interpret the underlying values and beliefs expressed through participants’ words and actions by delving into individuals’ experiences, emotions, behaviors, and the meanings behind social phenomena [3]. Common methods such as interviews, focus groups, and observations enable researchers to gather data that yield deep insights from diverse perspectives [4]. Qualitative analysis typically involves experienced researchers manually coding, categorizing themes, identifying patterns, and interpreting findings based on the research objectives [5]. This process requires significant time and effort because it relies heavily on researchers’ expertise and subjective judgments. While qualitative research provides rich and detailed data, its inherent complexity and resource-intensive nature pose significant challenges [2].

Recent advancements in artificial intelligence (AI) have highlighted its potential to revolutionize qualitative research by streamlining data management and supporting academic writing [6]. Large language models (LLMs) such as ChatGPT have emerged as promising tools for accelerating data analysis and improving the efficiency of qualitative coding [7]. By leveraging these tools, researchers can reduce the time required for conventional methods and analyze larger datasets [8].

However, ChatGPT’s architecture is predominantly based on English-language datasets, which account for over 90% of the training data [9]. This raises concerns regarding its applicability and accuracy when analyzing non-English languages, particularly languages such as Japanese, which differ significantly from English in linguistic structure [10]. Japanese presents unique challenges for AI-driven qualitative analysis due to its distinct linguistic and cultural characteristics. One major issue is the omission of subjects, which makes it difficult for AI to determine the actor in a given statement. In addition, the complex honorific system (keigo) encodes hierarchical relationships and politeness levels, which AI models may struggle to interpret accurately. Furthermore, indirect expressions and high context dependency in Japanese communication often require nuanced contextual understanding beyond the literal meaning. These factors contribute to the difficulty AI models face in accurately identifying themes within Japanese qualitative data [11]. Consequently, evaluating the utility of AI in this context is critical for understanding whether it can match the interpretative capabilities of human researchers or whether inherent limitations persist. Furthermore, assessing AI performance in analyzing Japanese-language interviews and data is vital for advancing qualitative research methodologies [12].

This study aimed to evaluate the effectiveness of generative AI tools, such as ChatGPT, in analyzing qualitative data in Japanese. Specifically, we analyzed interviews conducted in Japanese health care settings by comparing the thematic analysis results generated by ChatGPT with those produced by human researchers. In addition, we examined how linguistic and cultural nuances affect AI’s ability to perform qualitative analysis in non-English contexts. Thus, we aimed to elucidate the potential limitations of AI in qualitative research, ultimately contributing to the development of AI-assisted methodologies in this domain.

## Methods

### Research Design and Data Collection

Our research comprised a qualitative study that aimed to directly compare automated analysis using ChatGPT and manual analysis by human researchers to evaluate the utility and challenges of AI in qualitative data analysis. Verbatim transcripts from semistructured interviews conducted in Japanese were used as a dataset to assess the performance of AI analysis. In particular, we compared the AI and human analysis results in terms of theme consistency and depth of interpretation, focusing on the differences in cultural elements and emotional nuances. The agreement rates presented in this study are derived from relative frequency comparisons of themes identified by ChatGPT and human analysis. As the General Thematic Approach does not involve systematic frequency coding per interview, quantitative agreement metrics such as Cohen kappa were not applicable. Instead, we report descriptive agreement rates to provide a structured comparison.

The data were derived from semistructured interviews centered on experiences of “sacred moments.” The participants were 30 individuals, including health care professionals and patients, from an urban community hospital in Tokyo (Hospital A) and a rural university hospital (Hospital B). The interview guide was developed based on previous studies [13] (Multimedia Appendix 1).

### Interview Details

The interviews were conducted by experienced qualitative researchers, RS (General Medicine) and KS (Family Medicine). RS, a Japanese female with expertise in psychology and health-related research, has experience working at a Japanese university hospital. KS, a Japanese male, has a background in general medicine and public health, and has experience working at rural hospitals in Japan.

The interviews were conducted in a one-on-one format, with an average duration of 28.43 (range 16.75-48.00) minutes at Hospital A and 24.33 (range 15.42-40.20) minutes at Hospital B. All interviews were conducted in Japanese and focused on topics related to “sacred moments,” including participants’ experiences, the significance of such moments, and specific examples from medical practice. Transcripts were recorded verbatim (Multimedia Appendix 1). Participant selection criteria included intrinsic interest and experience related to the interview topics, with an emphasis on recruiting participants from diverse backgrounds to obtain a broad range of perspectives. The verbatim transcripts were transcribed using the automated NVivo (version 14; Lumivero) tool. Transcription accuracy was reviewed and the data were prepared for qualitative analysis.

#### Qualitative Data Analysis Using ChatGPT

We followed the established methods for using ChatGPT for qualitative data analysis [14]. The steps are outlined below.

#### Initial Analysis: ChatGPT (GPT-4o)

ChatGPT (GPT-4o) was used to analyze the qualitative data obtained from the interviews. ChatGPT was instructed to analyze each interview separately, following an approach that closely aligns with standard qualitative research practices [15]. In qualitative research, thematic analysis is typically conducted iteratively, where each interview is analyzed sequentially to assess data saturation. By adopting this approach, we aimed to mirror human thematic analysis as closely as possible without overemphasizing themes that appeared frequently in the initial interviews. The following prompts were provided to ChatGPT (Multimedia Appendix 2): “The text I just sent you is the transcript of an interview. Paragraphs starting with ‘A:’ were said by the interviewer, and paragraphs starting with ‘B:’ were said by the respondent. Now, please act like a researcher with expertise in qualitative research and thematically analyze this transcript.”

This initial prompt was applied 3 times to the same dataset to generate multiple responses, which were compared to evaluate consistency in the ChatGPT analysis. No additional instructions were provided to either human reviewers or ChatGPT during the initial thematic analysis. Human reviewers applied Braun and Clarke’s [15] six-phase approach independently, whereas ChatGPT analyzed the text solely based on the provided prompt without further modifications or guidance. The ChatGPT prompting was conducted by the first author, Kota Sakaguchi. The human reviewer (RS) independently performed thematic analysis without involvement in the ChatGPT analysis. ChatGPT was not provided with any additional information beyond the transcript text and the specified prompt. It did not receive contextual details about the interviewers, interviewees, or previous research. In contrast, the human reviewer (RS), who conducted the qualitative study, had previous knowledge of the interviewees based on direct interaction during the interviews.

#### Application of Alternative Analytical Approaches

A total of 2 widely recognized thematic analysis approaches in qualitative research were employed to assess the flexibility and consistency of the ChatGPT analysis (Multimedia Appendix 2). For each approach, the interview transcripts were loaded into a new ChatGPT session, and ChatGPT was instructed to analyze the data according to the respective method.

1. Grounded theory approach: Charmaz’s grounded theory was used, emphasizing the interaction between researchers and participants in collecting, interpreting, and reconstructing data [16]. ChatGPT was instructed as follows: “Please act like a researcher with expertise in qualitative research and analyze the transcript I have provided you with following the grounded theory approach proposed by Charmaz.”
2. Five-step framework approach: the five-step framework approach, which is widely used in qualitative research, was also applied. ChatGPT was instructed as follows [17]: “Please act like a researcher with expertise in qualitative research and analyze the transcript I have provided you with following the Five-Step Framework approach proposed by Pope and colleagues in 2000.”

These approaches were selected to evaluate the adaptability of ChatGPT to different thematic analysis methodologies.

### Analysis by Human Qualitative Researchers

To compare the results of ChatGPT’s analysis, the co-author RS (General Medicine) independently analyzed the interview transcripts without accessing ChatGPT’s output or prompts. The human researcher was provided with the same initial instructions provided to ChatGPT (“Please thematically analyze this transcript”) to allow for a flexible yet independent approach to analysis. Thematic analysis was conducted following the 6-phase approach proposed by Braun and Clarke [15]. First, the researchers familiarized themselves with the data by reading and re-reading the interview transcripts to gain an in-depth understanding. Second, initial codes were generated through the systematic coding of relevant features across the dataset. Third, similar codes were organized into potential themes. Fourth, themes were reviewed for coherence and refined to ensure consistency. Fifth, the final themes were clearly defined and named, refining their scope and specificity. Finally, the analysis was synthesized into a structured report, summarizing the key thematic findings. The human reviewer employed a General Thematic approach, which did not involve systematic frequency coding. This approach allows for greater flexibility in interpreting the meaning behind qualitative data without reducing the insights to numerical frequency counts. Cohen kappa could not be calculated due to the absence of data with structured presence or absence for each theme.

**Data Saturation and Reliability**

Data saturation was assessed throughout the analysis in accordance with the work by Hennink et al [18]. No new themes emerged after the 27th interview, suggesting that data saturation had been reached. To confirm the stability of thematic patterns, 3 additional interviews were analyzed, and the results remained consistent. To enhance the rigor and trustworthiness of the thematic analysis, we incorporated reflexivity, confirmability, dependability, credibility, and transferability as key methodological considerations. Reflexivity was ensured by maintaining a research journal throughout the analysis, allowing the researcher to critically reflect on potential biases. Confirmability was enhanced through systematic documentation of coding decisions and maintaining an audit trail. In addition, 20% of the interview data was independently recoded by a second researcher to assess reproducibility. Dependability was established through multiple coding consistency checks at different stages, with two independent researchers comparing their coding and resolving discrepancies through discussion. Credibility was reinforced by implementing a member-checking process, where selected participants reviewed preliminary findings to ensure the accuracy of identified themes. Transferability was considered by providing detailed descriptions of the data collection process and thematic categories, allowing researchers in other settings, particularly within East Asian medical contexts, to assess the applicability of the findings.

### Ethical Considerations

This study was approved by the institutional review board of Shimane University Hospital (approval number: KS20230706-1). As this study involved the secondary analysis of previously collected interview data, it was exempt from further ethical review under institutional guidelines. All participants were provided with a detailed explanation of the study objectives, procedures, and potential risks before their participation. Written informed consent was obtained from all participants, and no individuals who were recruited declined to participate. The informed consent explicitly allowed the reuse of collected data for further research purposes without requiring additional consent. To ensure privacy and confidentiality, all collected data were fully anonymized before analysis, and no personally identifiable information was included in the transcripts. The anonymized data were securely stored in a password-protected institutional repository, accessible only to the research team. Participants received a gift card worth 2000 JPY (approximately US $13) as compensation for their time and contribution to the study. No additional financial incentives or reimbursements were provided.

No images or figures included in this manuscript contain identifiable information about the participants. If any identifiable images were included, explicit consent from the individuals would have been obtained, and the relevant consent forms would have been submitted as supplementary materials.

## Results

This study conducted qualitative analyses of 30 Japanese interviews on “sacred moments” in clinical settings. The analyses compared thematic extractions conducted by ChatGPT with those conducted by experienced qualitative researchers. Furthermore, two distinct qualitative analysis approaches, the grounded theory and the five-step framework approach, were applied to evaluate the consistency and frequency of the extracted themes.

Tables 1 and 2 summarize the frequency and agreement rates of themes across the ChatGPT and human researcher analyses. The results demonstrate that ChatGPT identified major themes with relatively high consistency compared with human reviewers, particularly in frequently occurring themes such as “personal experiences of a sacred moment” and “strong connections between healthcare workers and patients.”

Themes identified in more than 25 (≥83%) interviews were observed with high frequency across analyses, whereas themes appearing in fewer than 9 (<30%) interviews were identified less consistently by both ChatGPT and human researchers. Thematic agreement was the highest for fundamental experiences commonly described in clinical settings, whereas less frequently mentioned themes (eg, “difficult to answer, no experience of sacred moments”) showed greater variability in identification rates.

These findings suggest that while ChatGPT demonstrates strong capabilities in detecting widely recurring qualitative themes, its performance in identifying less frequently mentioned or nuanced themes remains limited compared to human analysis. The results underscore the importance of human interpretative expertise in qualitative research, particularly for capturing the depth and complexity of subjective experiences.

**Table 1:** Themes identified through general thematic analysis^a^ (N=30).

Themes^b^	ChatGPT analysis		
	General thematic analysis #1, n (%)	General thematic analysis #2, n (%)	General thematic analysis #3, n (%)
1. Personal experiences of “sacred moments”^c^	28 (93)	30 (100)	28 (93)
2. Strong connections and emotionally stirring experiences between health care workers and patients	25 (83)	27 (90)	28 (93)
3. Building relationships	26 (86)	26 (86)	28 (93)
4. Shared-time	4 (13)	3 (10)	3 (10)
5. Dialogue	2 (6)	4 (13)	5 (16)
6. Perceived benefits for patients and health care workers	2 (6)	2 (6)	2 (6)
7. Elements needed to build trust	2 (6)	3 (10)	3 (10)
8. Showing interest in others, getting to know them deeply	2 (6)	2 (6)	2 (6)
9. Difficult to answer, no experience of sacred moments	1 (3)	1 (3)	1 (3)
10. Fate	0 (0)	0 (0)	0 (0)

^a^Themes identified through General Thematic Analysis from interviews conducted in Japan from October to November 2024, centered on the theme of “sacred moments” in health care settings. The study involved qualitative interviews with health care providers and patients, and the table compares themes extracted by a qualitative researcher and ChatGPT, indicating the frequency of each theme across 30 interviews.

^b^Themes were ordered to highlight similarities and differences across analytic approaches.

^c^The first author edited theme names in partnership with the human researcher to bolster clarity. The original theme titles and descriptions are available in Multimedia Appendix 3.

**Table 2:** Themes identified through grounded theory and framework approach^a^ (N=30).

Themes^b^	ChatGPT analysis			
	Grounded theory, n (n%)	Framework approach, n (n%)		
1. Personal experiences of “sacred moments”^c^	22 (73)	25 (83)		
2. Strong connections and emotionally stirring experiences between health care workers and patients	21 (70)	22 (73)		
3. Building relationships	26 (86)	25 (83)		
4. Shared-time	8 (26)	8 (26)		
5. Dialogue	4 (13)	4 (13)		
6. Perceived benefits for patients and health care workers	3 (10)	3 (10)		
7. Elements needed to build trust	4 (13)	3 (10)		
8. Showing interest in others, getting to know them deeply	3 (10)	3 (10)		
9. Difficult to answer, no experience of sacred moments	1 (3)	2 (6)		
10. Fate	0 (0)	0 (0)		

^a^Themes identified using grounded theory and framework approach in the same dataset of interviews conducted in Japan from October to November 2024, centered on the theme of “sacred moments” in health care settings. The table provides a comparative analysis of themes identified by a qualitative researcher and ChatGPT, showing their relative occurrence across 30 interviews.

^b^Themes were ordered to highlight similarities and differences across analytic approaches.

^c^The first author edited theme names in partnership with the human researcher to bolster clarity. The original theme titles and descriptions are available in Multimedia Appendix 3.

### Extraction of Common Themes: Frequency and Agreement Rates

Among the extracted themes, high consistency was observed for “personal experience of a sacred moment” (28/30, 93%), “strong connections and emotionally stirring experiences between healthcare workers and patients” (28/30, 93%), and “building relationships” (28/30, 93%). These themes exhibited agreement rates exceeding 80% between ChatGPT and human researchers, suggesting that ChatGPT demonstrates strong capabilities in identifying recurrent themes in qualitative data, even in Japanese-language contexts. Furthermore, the reproducibility of these findings emphasizes the potential utility of ChatGPT as a supportive tool for qualitative research.

### Limitations in Analyzing Cultural Elements

Conversely, themes with strong cultural and emotional connotations, such as “difficult to answer, no experience of sacred moments” and “fate,” were identified in fewer than 3 interviews (<10%) and exhibited agreement rates below 30%. These results highlight ChatGPT’s limitations in interpreting Japanese-specific sociocultural expressions and implicit emotional nuances. To enhance interpretability, we have supplemented these themes with explicit definitions and representative example sentences. For instance, the theme of “fate” was characterized by statements where participants attributed life events or medical outcomes to forces beyond human control. An example statement includes: “No matter how much we try, some things are determined by fate.” This refinement enhances the transparency and objectivity of our results.

### Results From the Grounded Theory Approach

Using the grounded theory approach, certain themes demonstrated high occurrence and agreement rates, including “personal experience of a sacred moment” (22/30, 73%), “strong connections and emotionally stirring experiences between healthcare workers and patients” (21/30, 70%), and “building relationships” (26/30, 86%). These results suggest that ChatGPT can identify fundamental thematic elements that emerge consistently across different analytical frameworks. However, other themes, such as “dialogue” (4/30, 13%) and “fate” (0/30, 0%), showed lower frequency and agreement, particularly for culturally and interpretatively rich topics.

### Results From the Five-Step Framework Approach

Similarly, under the five-step framework approach, high frequency and agreement rates were observed for “personal experience of a sacred moment” (25/30, 83%), “strong connections and emotionally stirring experiences between healthcare workers and patients” (22/30, 73%), and “building relationships” (25/30, 83%). These findings reinforce the trend observed in the grounded theory approach, indicating that ChatGPT consistently recognized themes that were explicitly described within the interview data. In contrast, themes requiring deeper interpretation, such as “benefits for patients and healthcare providers” (3/30, 10%), “difficult to answer, no experience of sacred moments” (2/30, 6%), and “fate” (0/30, 0%), exhibited significantly lower frequency and agreement rates. These results emphasize ChatGPT’s limitations in analyzing nuanced and culturally embedded themes, which require deeper contextual understanding beyond surface-level textual analysis.

## Discussion

### Principal Findings

In this study, we analyzed and compared 30 Japanese interviews focusing on “sacred moments” in clinical settings using ChatGPT and human researchers. Themes such as “personal experience of a sacred moment,” “strong connections and emotionally stirring experiences between healthcare workers and patients”, and “building relationships” were identified in over 83% of interviews and exhibited high agreement rates (≥83%) between ChatGPT and human researchers. These findings indicate that ChatGPT possesses strong capabilities in extracting frequently occurring themes within Japanese-language qualitative data, supporting its potential as a supplementary tool for qualitative research [19].

Conversely, themes deeply embedded in sociocultural contexts, such as “difficult to answer, no experience of sacred moments” and “fate,” demonstrated agreement rates below 30%, highlighting the limitations of AI in interpreting culturally and emotionally nuanced expressions in Japan. To improve the transparency of these findings, we supplemented key themes with explicit definitions and example sentences. For instance, the theme of “fate” was characterized by statements in which participants attributed life events or medical outcomes to forces beyond human control. An example statement includes: “No matter how much we try, some things are determined by fate.” This refinement enhances the interpretability of the results [20]. While ChatGPT’s ability to quantify themes provides a structured numerical comparison, human thematic analysis inherently emphasizes interpretative depth. This methodological difference should be considered when evaluating the comparative outputs of AI-assisted and human-led qualitative research.

### Comparison With Previous Studies

These results align with previous studies that identified the strengths of ChatGPT in extracting descriptive themes [19,21]. Notably, its ability to achieve high agreement rates for descriptive themes in non-English languages underscores the potential of LLMs to support qualitative analyses across multiple languages [22]. Recent studies, such as those conducted by Pattyn [23], have explored the application of generative AI models in qualitative research. While these studies demonstrate AI’s potential in automating thematic identification and data synthesis, key challenges remain, including the risk of overgeneralization, context misinterpretation, and ethical concerns regarding AI-generated analyses. Our study extends this line of research by systematically comparing ChatGPT’s performance against human thematic analysis in a linguistically and culturally complex context, highlighting both its capabilities and its inherent limitations [24].

Future research should continue to explore best practices for integrating AI into qualitative methodologies, ensuring rigor, interpretability, and contextual accuracy. Furthermore, previous research has suggested that AI models trained primarily on Indo-European languages may struggle with Asian language structures [25]. Jalali and Akhavan [22] explored AI’s role in multi-language qualitative analysis and demonstrated its potential in thematic identification across diverse linguistic contexts. However, these studies primarily focused on European and Indo-European languages, which differ structurally from Japanese. Our study contributes to this body of research by assessing AI’s performance in analyzing qualitative data presented in Japanese, a language characterized by subject omission, honorifics, and indirect expressions [26]. This distinction highlights the need for further investigation into AI’s adaptability across linguistically complex settings, particularly in East Asian contexts [22].

### Strengths and Limitations

While this study demonstrates valuable insights, several limitations must be acknowledged.

First, the study sample consisted of 30 interviews, which provides exploratory insights but limits broad generalizability. However, data saturation was reached after analyzing the 27th interview, and subsequent interviews confirmed the consistency of thematic patterns, thereby suggesting that key themes were well captured. Given that qualitative research prioritizes depth and rich contextual understanding over large-scale representativeness, our findings serve as a foundational step for future studies that may apply larger sample sizes across diverse health care settings to validate and extend these insights [20].

Second, this study focused exclusively on ChatGPT (GPT-4). Advancements in AI language models, such as GPT-4o and GPT-o1, may offer improved performance in analyzing culturally nuanced qualitative data. These newer models incorporate enhanced multilingual capabilities, improved contextual understanding, and greater adaptability to complex linguistic structures such as honorifics, subject omission, and indirect expressions in Japanese [27]. Future studies should explore comparative evaluations of these models to assess their potential in refining AI-assisted thematic analysis [28].

Third, limitations exist in ChatGPT’s ability to analyze deeply cultural and emotional themes. While most of the themes identified by ChatGPT aligned with those recognized by human researchers (themes 1-4), some themes (themes 5 and 6) required minor renaming to ensure consistency with human-coded themes. These adjustments were made to improve comparability while preserving the core meaning of the themes [29].

Fourth, methodological constraints should be considered. We recognize that future studies could explore structured coding schemes, such as Framework Analysis, to enable the calculation of interrater reliability metrics like Cohen κ or Krippendorff α. However, in this study, the focus was on interpretative thematic analysis rather than numerical coding [30].

Fifth, ethical and privacy concerns remain critical considerations in AI-assisted qualitative research. Although all data were anonymized and securely stored, further discussions are needed regarding data protection frameworks, algorithmic biases, and the responsible implementation of AI in qualitative analysis [31].

### Directions for Future Research

To further refine AI-driven qualitative analysis in Japanese, future research should focus on developing structured AI models adapted to the linguistic and cultural characteristics of Japanese. Collaborations between computational linguists, AI researchers, and domain experts could facilitate the construction of a Japanese-specific corpus tailored for thematic analysis. Adjusting the model architecture to incorporate context-aware processing mechanisms would enhance AI’s ability to interpret nuanced conversations [32]. Expanding training datasets to include diverse Japanese-speaking communities can further improve the robustness of AI-assisted qualitative analysis [33]. While statistical agreement measures could strengthen future comparisons between AI and human qualitative analysis, our study primarily focuses on conceptual consistency rather than numerical agreement. Future studies employing structured coding frameworks may facilitate statistical assessments such as the McNemar test or chi-square analysis.

In addition, as AI capabilities continue to evolve, comparative studies of newer models should be conducted to assess whether they offer improved interpretability and accuracy in non-English qualitative research [28]. Finally, a more extensive exploration of hybrid AI-human analytical frameworks may be beneficial in leveraging the strengths of both AI-driven automation and human interpretative depth [34]. To further validate the cultural specificity of extracted themes, future studies could involve independent expert reviewers who are blinded to the source (AI vs human). These reviewers would assess whether the themes represent culturally unique or globally universal concepts. Such external evaluations may enable quantifiable comparisons between AI- and human-generated analyses regarding cultural sensitivity.

### Conclusions

This study demonstrated that ChatGPT possesses a high capability for extracting descriptive themes from Japanese qualitative data while revealing its limitations in addressing themes requiring cultural context and deep interpretation. These findings suggest a supplementary role for AI in qualitative research, emphasizing the continued importance of human researchers’ insights. Future research should focus on evaluating the applicability of AI across different cultural and linguistic contexts and address the ethical considerations associated with its use. Note: Language editing was supported by ChatGPT-4.0 (OpenAI) to enhance clarity and readability. All AI-assisted outputs were reviewed and validated for accuracy by the authors prior to submission.

## Appendix

Multimedia Appendix 1 Analysis by ChatGPT based on General thematic analysis.

Multimedia Appendix 2 Analysis by ChatGPT based on Grounded Theory.

Multimedia Appendix 3 Analysis by ChatGPT based on Five Step Framework Approach.

## Abbreviations

AI: artificial intelligence
LLM: large language model

## Data Availability

The data used in this study are available from the corresponding author upon reasonable request.
