# Supplementary figures and images for: Evaluating ChatGPT in Qualitative Thematic Analysis With Human Researchers in the Japanese Clinical Context and Its Cultural Interpretation Challenges: Comparative Qualitative Study
Source: J Med Internet Res. 2025 Apr 24;27:e71521. doi: 10.2196/71521 (PMC12062757; doi:10.2196/71521)

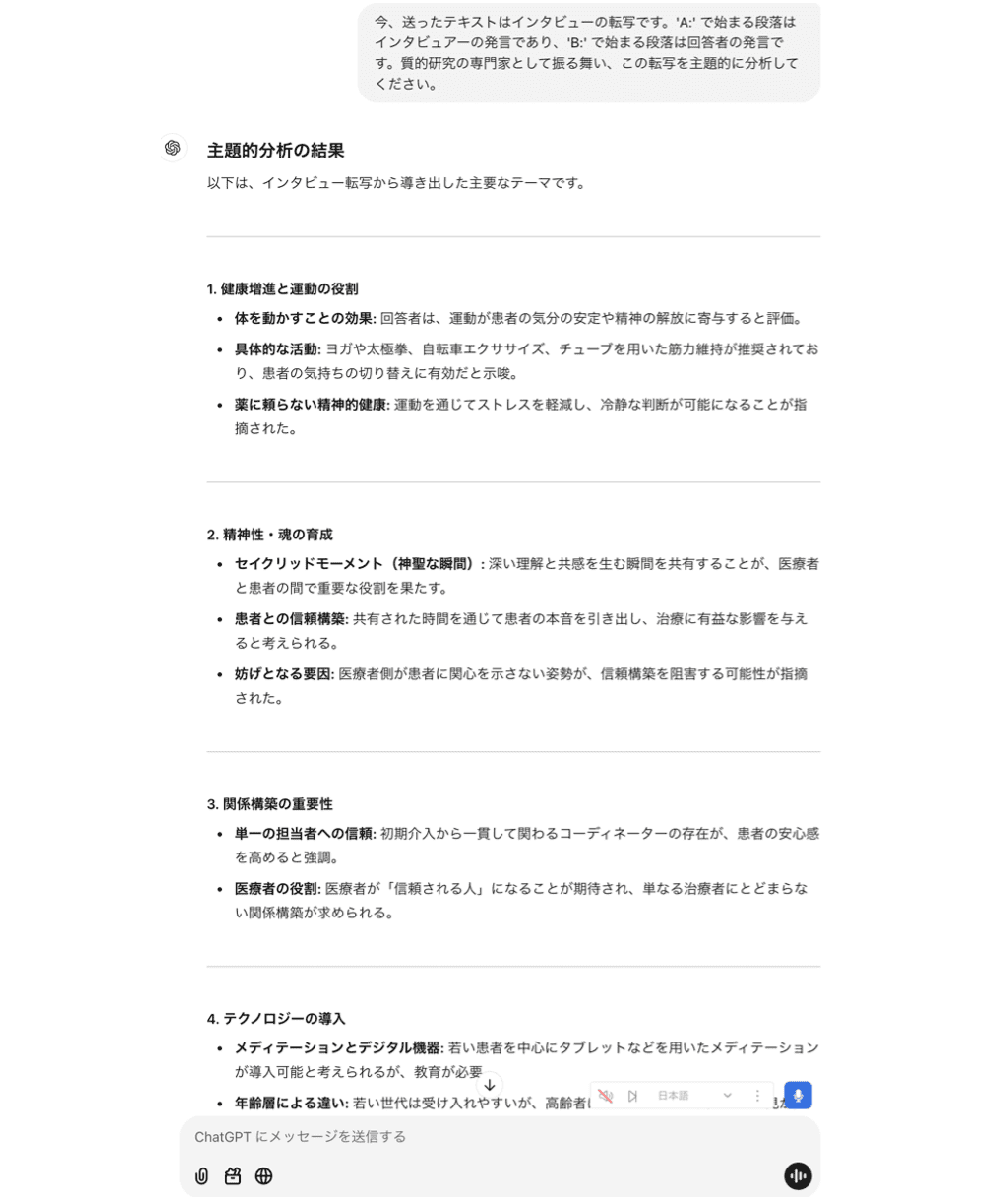

Supplement: Multimedia Appendix 1 [file jmir_v27i1e71521_app1.png]

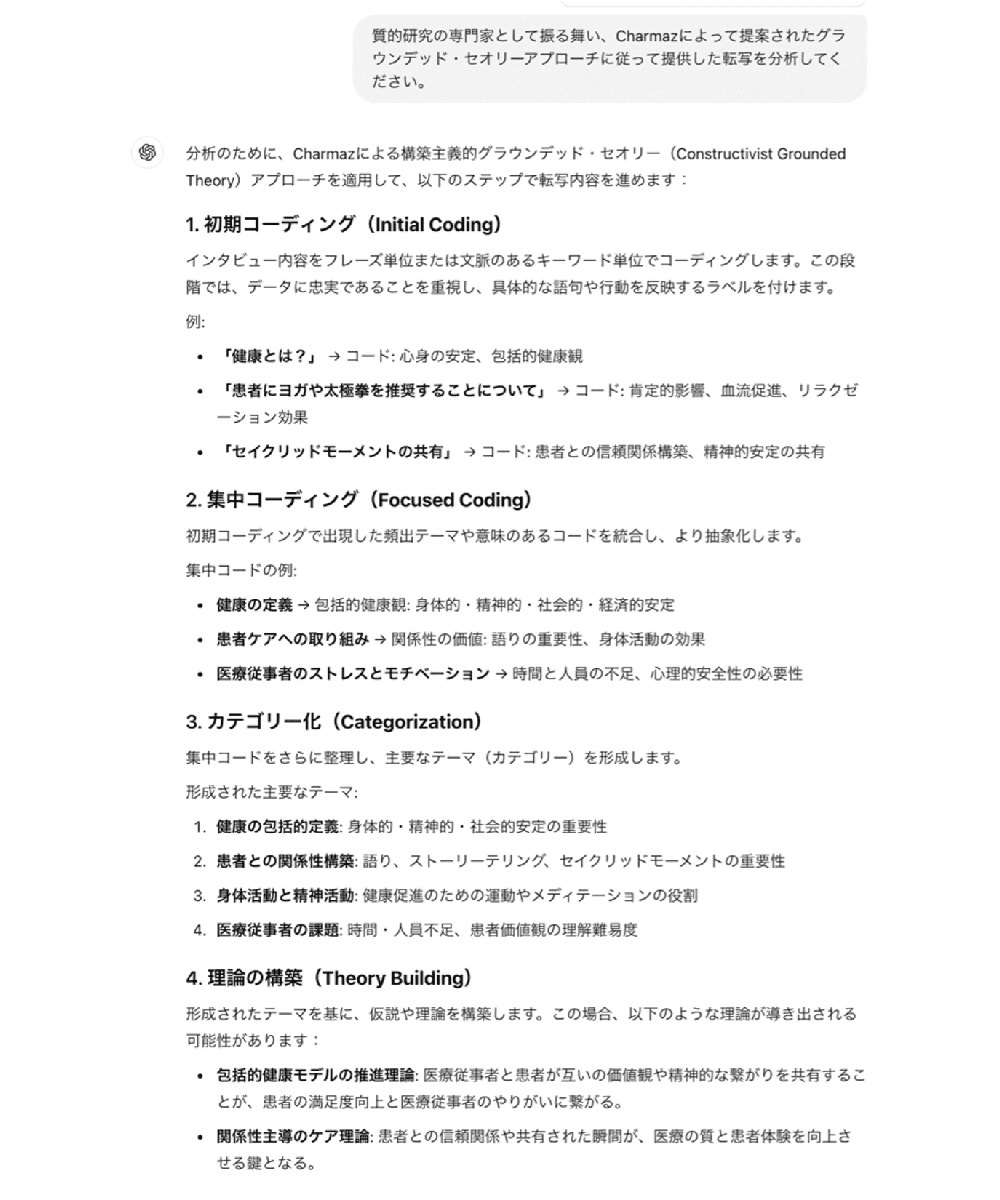

Supplement: Multimedia Appendix 2 [file jmir_v27i1e71521_app2.png]

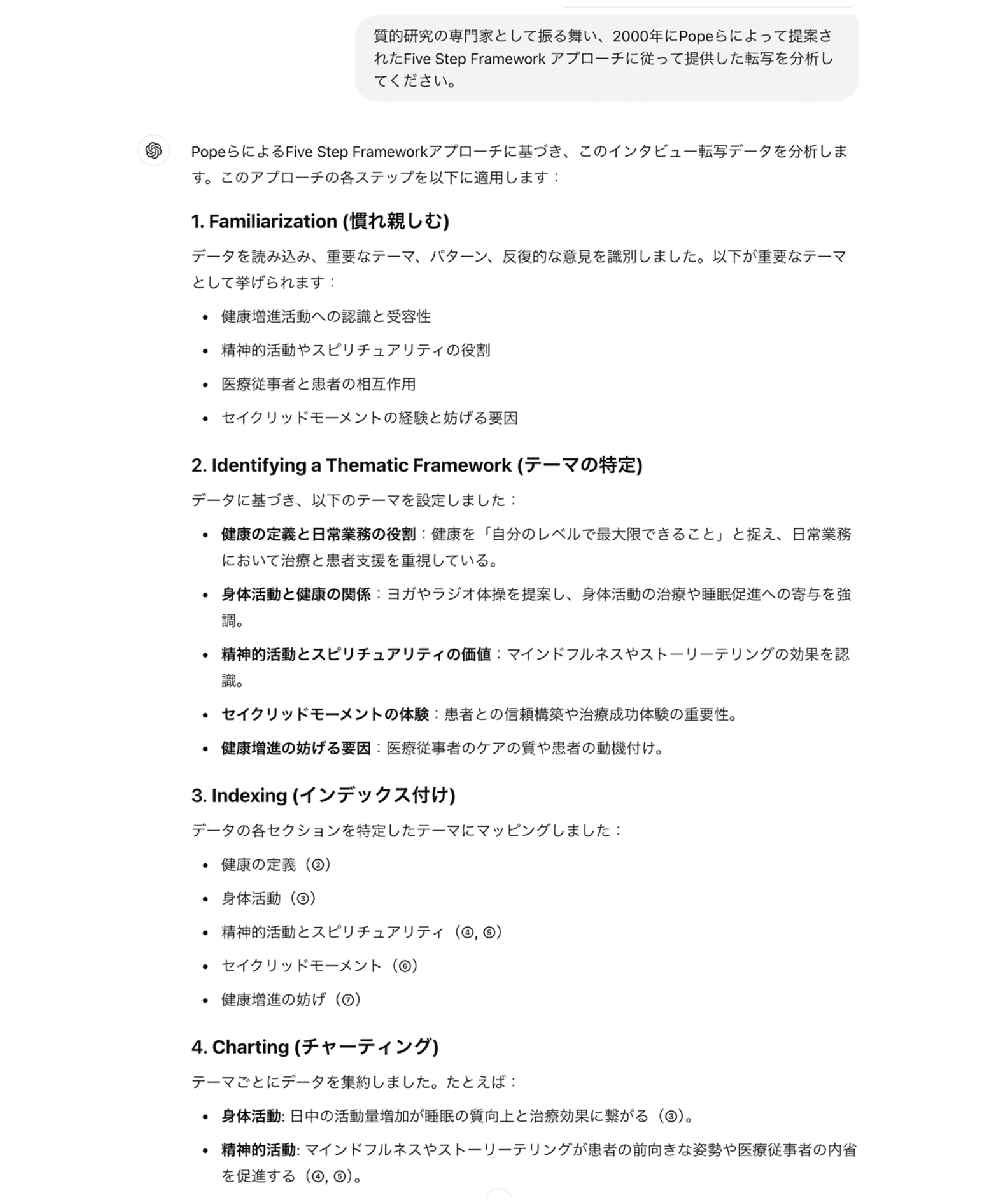

Supplement: Multimedia Appendix 3 [file jmir_v27i1e71521_app3.png]
